# Intracranial inflammatory pseudotumour related to IgG4: A very rare case

**DOI:** 10.22088/cjim.15.2.354

**Published:** 2024

**Authors:** Ghassen Gader, Meissa Hamza, Ftima Jaziri, Ines Chelly, Ihsèn Zammel, Mouna Rkhami, Mohamed Badri

**Affiliations:** 1 Department of Neurosurgery, Trauma and Burns Center, University of Tunis-El Manar, Faculty of Medicine of Tunis, Ben Arous, Tunisia; 2 Department of Internal Medicine, Sadok Mokaddem Hospital of Jerba, Ben Arous, Tunisia; 3 Department of Pathology, La Rabta Hospital, University of Tunis-El Manar, Faculty of Medicine of Tunis, Tunis, Tunisia

**Keywords:** Inflammatory pseudotumor, IgG4, Neurosurgery, Internal Medicine

## Abstract

**Background:**

Intracranial inflammatory pseudotumours (IPT) are rare entities that frequently lead to misdiagnosis with malignant lesions. The identification of these lesions is difficult, but important to avoid inadvertent iatrogenicity and to adjust therapeutic protocols.

**Case Presentation:**

We report the case of a 30-year-old man who presented a single tonic-clonic seizure. Brain imaging showed a right frontal lesion with intra and extra axial components. Facing the radiologic presentation, a brain tumor was suspected, thus the patient underwent surgery. Pathological exam concluded to a plasma cell granuloma. A whole-body CT-scan showed only a thoracic aortitis. Complete blood work studies came back negative. The patient was also tested for an array of antibodies among which antinuclear antibodies were positive (blood level superior to 1/100). CSF evaluation revealed clear fluid with normal glucose concentration, normal protein levels and lymphocytic pleocytosis. Finally, IgG-4 plasma levels were elevated which led to the diagnosis of an IgG4-RD. The patient was put under prednisolone with a favorable outcome.

**Conclusion:**

IPT have several etiologies, among which IgG4 related disease may be one of the less known as only 2 cases have previously been reported. Herein, we report a new case of a young man who presented for seizures related to an intracranial lesion of an IgG4 related disease. The challenge is to suspect such conditions to avoid unnecessary surgeries.

Intracranial inflammatory pseudotumors (IIPT) are among the differential diagnosis for central neurologic system (CNS) neoplasms. Despite their rarity, they may cause diagnostic issues as they can clinically and radiologically mimic intracranial tumors (1). They still remain of an unclear etiopathogeny. IIPT are mainly related to sarcoidosis and Histicytosis (Rosaï Dorfman disease) (1, 2). Intracranial localization for IgG-4 related disease (IgG4-RD) is very rare, as to the best of our knowledge, only two cases of IgG4 related intracranial inflammatory pseudotumors were reported to date within brain parenchyma (2). IgG4-RD was long considered as a single organ disease until 2003, when Kamisawa (3) suggested the clinicopathological entity ‘’IgG4-*related autoimmune disease’’. *A new understanding of this disease came to light. 

In point of fact, IgG4-related disease (IgG4-RD) is an immune mediated fibro-inflammatory disease of unknown cause characterized by the infiltration of one or more target organs by IgG4-positive plasma cells and lymphocytes (4–6). Although it mostly affects digestive organs and salivary and lacrimal glands, a variety of central nervous system (CNS) manifestations are now recognized. These CNS manifestations are quite uncommon, among which hypophysitis and hypertrophic pachymeningitis are the most frequently reported lesions (2, 7). We herein report the third case of a recently admitted patient with a parenchymal IgG4- related inflammatory pseudotumor.

## Case Presentation

**Figure 1:**
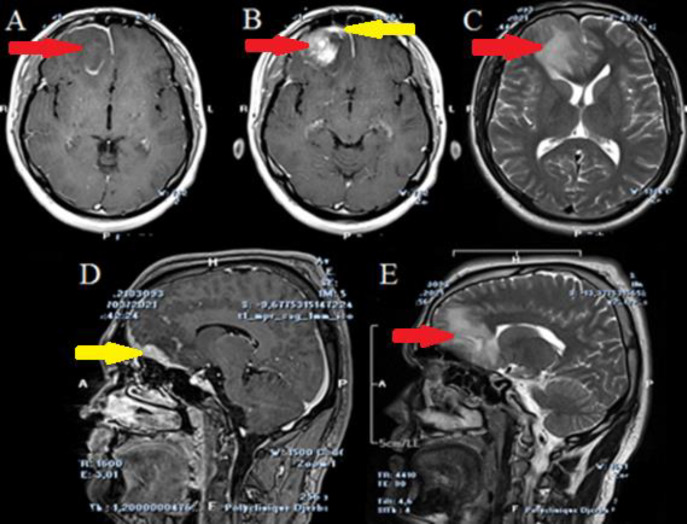
Axial section (A, B, C) and sagittal section (D, E) of a brain MRI on T1-WI without Gadolinium (A), with Gadolinium (B,D), T2-WI (C, E) of a preoperative MRI showing a right frontal lesion with a heterogeneous enhancement surrounded by finger like edema. Sagittal reconstructions show right fronto-basal pachymeningitis

We report the case of a 30-year-old man with no pathologic background, who presented a single tonic-clonic seizure four months ago. He complained of neither intracranial hypertension symptoms, nor visual symptoms. Neurological and physical exam were normal. Brain CT scan (figure 1) showed a right frontal lesion with intra and extra axial components. Brain MRI showed that the extraxial lesion was related to a hypertrophic pachymeningitis underlaying an intraparenchymal mass which showed a heterogenous enhancement following injection of Gadolinium. A finger-like edema signal was surrounding. At this stage, we mainly suspected a CNS lymphoma or a glioma, but the underlying pachymeningitis was against this hypothesis. The decision was to operate the patient for the most complete possible resection and to obtain the pathologic confirmation. Peroperative, the intra-axial lesion was greyish, non-hemorrhagic, and came easily into suction. There was neither necrosis nor thrombosed veins. The pachymeningitis was extensive and had a meningioma-like aspect. We entirely removed the intra-axial portion, whereas the pachymeningitis was extending towards the fronto-basal region, thus inaccessible through our surgical corridor.

**Figure 2:**
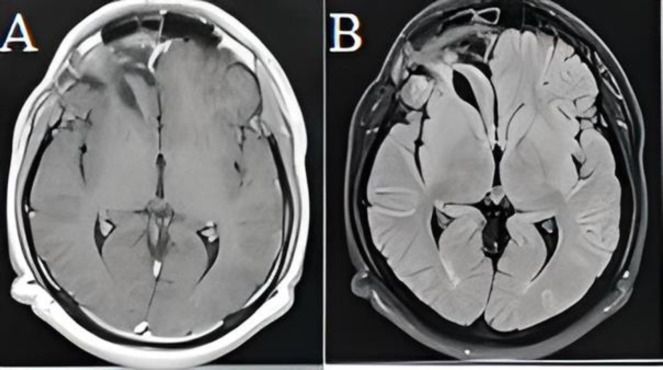
Axial sections of a brain MRI on T1-WI (A) and T2-DWI (B) showing a complete regression of the lesion

**Figure 3:**
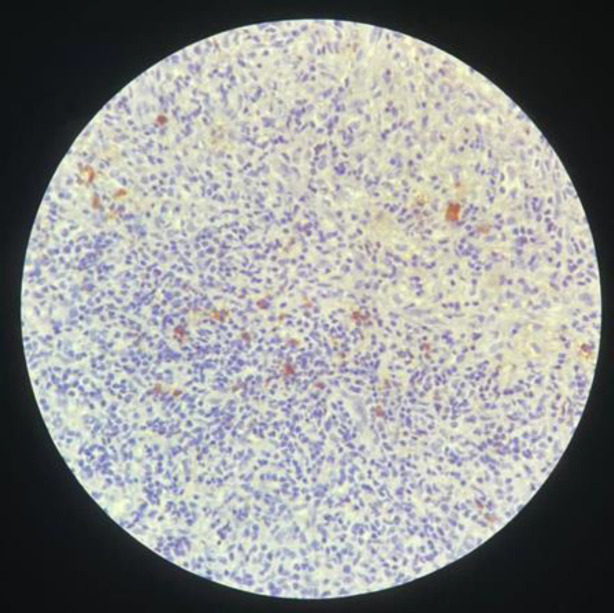
Histopathological findings of the mass resected showing massive infiltration of lymphocytes and plasma cellson H &E staining. IgG4 immunostaining shows > 10 IgG4 + plasma cells/HPF

Postoperative course was uneventful, and control CT scan (figure 2) showed no complications. The patient was discharged at the 4^th^ postoperative day. Pathological exam (Figure 3) concluded to a plasma cell granuloma. A malignant blood disorder became the first working diagnosis, followed by infectious and inflammatory diseases. A whole-body CT-scan showed no more than thoracic aortitis. Complete blood work studies, including serology of infectious diseases, tumor markers screenings, anti-NMO antibodies and vasculitis tests were performed and came back negative. 

The patient was also tested for an array of antibodies among which antinuclear antibodies were positive (blood level superior to 1/100). A lip biopsy was also performed and showed no abnormalities. CSF evaluation revealed clear fluid with normal glucose concentration, normal protein levels and lymphocytic pleocytosis. Finally, IgG-4 plasma levels were elevated to 2g/L (normal values vary between 0.040 and 0.870 g/L) which led to the diagnosis of an IgG4-RD. The patient was put under 0.6mg/kg/d of prednisolone during 15 days followed by a progressive degression over 6 months. One year after surgery, the patient showed neither clinical nor radiological signs of recurrence.

Red Arrows: Intra-axial lesions; Yellow Arrows: Extra-axial lesions

## Discussion

IgG4-RD is a rare auto-immune disease that was first identified in 2001, initially been considered as responsible for chronic pancreatitis (3). By 2003, the condition had been reclassified as a systemic disease most commonly affecting the pancreas, salivary and lacrimal gland. IgG4 represents less than 5% of the total IgG pool in healthy individuals. Although its high serum level is one of the diagnostic criteria, there is growing acceptance that IgG4 itself is unlikely to be pathogenic in IgG4-RD (6). Hyperproduction of plasmablasts (CD19+CD20−CD27+CD38+) may correlate more strongly with disease activity than IgG4 levels. How and why specific B cells expand into IgG4-producing plasmablasts and plasma cells is still to be eluded, but T follicular helper cells appear to drive the class switch towards IgG4, potentially through the secretion of IL-4 (8). Various etiologies seem to be such as trauma, infection (EBV, bacteria, fungi, etc), abnormal immune response, as well as a genetic support, as an ALK gene rearrangement in 2p23 was found in 50% of the cases (9–11). Few large-scale epidemiological studies of IgG4-RD have been performed. Most of these studies report male predominance, while it is a female predominance in other auto-immune diseases (8, 11). The mean age varies between 53 and 69 years. Even in the cases of multi-organ involvement, clinical presentation is typically dominated by a one organ symptoms.

The most common CNS manifestations of IgG4-RD are related to hypertrophic pachymeningitis and hypophysitis (6, 7, 10). Pachymeningitis in IgG4 RD can be cerebral, spinal, or rarely both. 8.8% of pachymeningitis cases are related to IgG4-RD. Symptoms vary whether the lesion of the dura is diffuse or localized: from cranial palsies by focal mechanical compression to headaches, seizures and cognitive decline by a more widespread involvement. IgG4-related hypophysitis most often presents with panhypopituitarism (11) with unspecific symptoms. To the best of our knowledge, this is the third reported case of IgG4-RD related IPT of cerebral parenchyma (table 1). 

**Table 1:** Summary of the previously reported cases of intracerebral lesions related to IgG4 disease

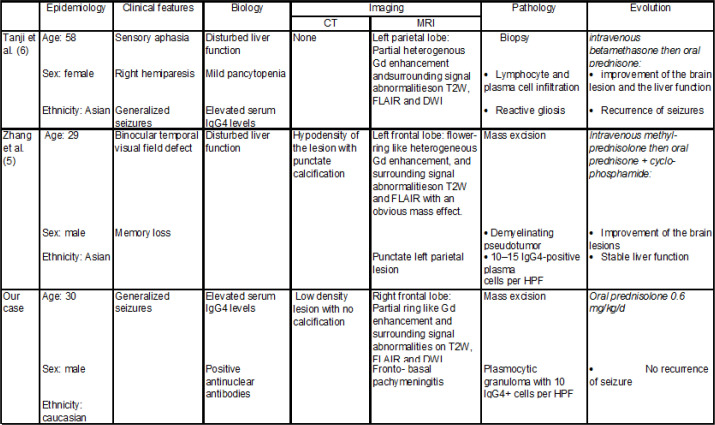

Imaging is essential for topographic study, but does not provide any specificities that may facilitate differential diagnosis. Radiologic findings of cerebral IPT may be found, namely an infiltrative parenchyma mass lesion that can be surrounded by edema. On CT, the enhancement pattern flower-ring may mimic a high-grade glioma but, the cellular architecture of these malignant tumors tends to make them iso- to hyperdense on CT and exempt of calcifications while IPTs, as the name indicates, have an inflammatory arrangement that gives an iso- to hypodense aspect with or without calcifications (2). The characteristics on the CT images of our case revealed a hypodense lesion with no calcification but with a flowering enhancement. The psammoma bodies of IPT (or plasma-cellular granuloma) can also be distinguished by their histopathological features which suggests that calcification is an important criterion in the imaging

Most of literature findings on imaging treats of IPT mimicking meningioma: these lesions are iso intense in T1-WI and T2-WI, and show intense enhancement and interdigitations with adjacent cortex following injection, suggesting local infiltration so that the most common preoperative radiological diagnosis is meningioma if single, and metastases if multiple (9). In our case, there was an isolate intense enhancement on T1WI of the underlying thickened dura suggesting pachymeningitis, but the intraparenchymal lesion was hypointense on T1-WI, of heterogenous signal on T2-WI with a ring-like enhancement pattern. We considered the differentials of a CNS lymphoma (backed up by the signal-intensity recovery study) and a high-grade glioma considering spectroscopy data. The pachymeningitis could be a reactive thickening by infiltration or, an argument in favor of an inflammatory disease.

In the last decade, 2-[18F]-fluoro-2-deoxy-D-glucose positron emission tomography–computed tomography (FDG PET-CT) has emerged as a useful tool in the diagnosis and monitoring of IgG4-RD. FDG uptake is correlated with the disease activity and improves after treatment (12). Histopathological examination is the key to diagnosis. Lymphoma and cell-rich meningiomas are the main differential diagnoses that might be difficult to distinguish from inflammatory pseudo tumors. Lymphoma should be suspected when the inflammatory components dominate and the inflammatory pseudotumor shows evidence of polyclonality in the mononuclear population (10). There are also previous reports of IgG4-related diseases in which an occult lymphoma was later revealed (7). Histopathological features most specific for IgG4-RD are lymphoplasmacytic infiltrates, storiform fibrosis, and venulitis. The predominance of each of these patterns may vary based on the involved organ. Once at least two of the three morphological criteria have been met, immunohistological staining of the cells is extremely useful for solidly classifying these lesions within the IgG4-related diseases. Another tool of particular importance is CSF analysis which offers the possibility to make a positive diagnosis, and may also give specific information about intrathecal synthesis of IgG4 (13,14). In our case, as the patient presented with seizures related to an intracranial mass out of any context of systemic disease, we decided to operate for resection, and to obtain samples for pathologic study. Later, and adding all arguments collected through different investigations, the diagnosis was held following the Japanese criteria (4) that state:

1. Characteristic diffuse/localized swelling or masses in single or multiple organs: our patient presented with a brain parenchyma mass associated to localized pachymeningitis.
2. Hematological examination showing elevated serum IgG4 concentrations (1,35 g/L) whereas it was up to 2g/L in our patient’s serum
3. Histopathological examination showing: .a. marked lymphocyte and plasmacyte infiltration and fibrosis, .b. infiltration of IgG4 + plasma cells: ratio of IgG4+/IgG + cells > 40% and > 10 IgG4 + plasma cells/HPF. Definite diagnosis is held if all of the conditions are met, probable if 1 + 3 and possible if 1 + 2. This case was compatible with a diagnosis of definite IgG4-RD.

The prognosis of intracranial inflammatory pseudo tumors is serious. This tumor is considered a benign entity, but neoplastic transformation with aggressive growth has also been reported (15). Monitoring serum IgG4 levels and circulating plasmablasts provide information on the active status of the disease (8). No consensus regarding treatment schedules has been yet established due to the rarity of the condition (2, 9–11, 16). The ongoing protocols come from systematic reviews of literature and expert guidance (17). The watchful wait could be indicated in some patients, but if a vital organ is involved, immediate and aggressive treatment is required (6). 

Some patients need urgent surgical intervention: cranial nerve decompression, laminectomy , etc. (8, 11). But the main treatment remains pharmacological. Glucocorticoids are the first line treatment. The clinical response is excellent but unsustained (8). It is crucial to evaluate the extent of fibrosis in the involved organ: IgG4-RD often progresses from lymphoplasmocytic inflammation to extensive fibrosis: a stage at which response to treatment is less likely (6, 17). Response is usually recorded within two weeks: glucocorticoids are 97% effective but with a relapse rate estimated at 33%. Relapse treatment falls under increased dosage (16). Although it is common to usually start with a dose of 30-40mg/day, neurological IgG4-RD require an initial course of intravenous methylprednisolone, 500-1000mg a day for 3 days in attempt to rapidly limit irreversible CNS damage. Authors suggest to maintain treatment up to three years if possible, given a 3 – years recurrence rate at 92% according to some studies (8). It is safe to consider patients under treatment as remitting rather than cured since IgG4-RD follows a relapsing-remitting course as well as a metachronous fashion recruiting different organs over the time (3)

Chemotherapy agents such as aziathroprine, mycophenolate mofetil, methotrexate (MTX) among others have been used as remission maintenance drugs or as first line treatment if there is a contraindication to steroids. Their efficacy requires further evaluation (17). MTX was reported to give conflicting results with neurological IgG4-RD (7). B cell depletion strategy using Rituximab (RTX) has promising results with patients’ refractory to glucocorticoids or presenting relapses. It enabled rapid glucosteroid tapering as well as encouraging clinical, radiological and serologic responses (17). 

These protocols have been discussed for systemic IgG4-RD without any specificities for CNS involvement. In our patient, as well in the previous experiences, intracranial lesions seemed to be controlled under corticosteroids used in "conventional" dosage.

We believe that this report emphasizes the need to maintain a high-level clinical vigilance towards the myriad of clinical and radiological manifestations of IgG4-RD and CNS IPT in general that may lead to an unfortunate misdiagnosis and or an unnecessary surgery. Neurological involvement may occur at presentation or upon follow up. 
